# Endoplasmic Reticulum Plays a Critical Role in Integrating Signals Generated by Both Biotic and Abiotic Stress in Plants

**DOI:** 10.3389/fpls.2019.00399

**Published:** 2019-04-04

**Authors:** Chang-Jin Park, Jeong Mee Park

**Affiliations:** ^1^ Department of Bioresources Engineering, Sejong University, Seoul, South Korea; ^2^ Plant Engineering Research Institute, Sejong University, Seoul, South Korea; ^3^ Plant Systems Engineering Research Center, Korea Research Institute of Bioscience and Biotechnology (KRIBB), Daejeon, South Korea; ^4^ Department of Biosystems and Bioengineering, University of Science and Technology (UST), Daejeon, South Korea

**Keywords:** ABA, ER stress, heat stress, osmotic stress, RNA virus, SA

## Abstract

Most studies of environmental adaptations in plants have focused on either biotic or abiotic stress factors in an attempt to understand the defense mechanisms of plants against individual stresses. However, in the natural ecosystem, plants are simultaneously exposed to multiple stresses. Stress-tolerant crops developed in translational studies based on a single stress often fail to exhibit the expected traits in the field. To adapt to abiotic stress, recent studies have identified the need for interactions of plants with various microorganisms. These findings highlight the need to understand the multifaceted interactions of plants with biotic and abiotic stress factors. The endoplasmic reticulum (ER) is an organelle that links various stress responses. To gain insight into the molecular integration of biotic and abiotic stress responses in the ER, we focused on the interactions of plants with RNA viruses. This interaction points toward the relevance of ER in viral pathogenicity as well as plant responses. In this mini review, we explore the molecular crosstalk between biotic and abiotic stress signaling through the ER by elaborating ER-mediated signaling in response to RNA viruses and abiotic stresses. Additionally, we summarize the results of a recent study on phytohormones that induce ER-mediated stress response. These studies will facilitate the development of multi-stress-tolerant transgenic crops in the future.

## Introduction

Being sessile organisms, plants rely on their interactions with various organisms to adapt to environmental changes. Most terrestrial plants establish symbiotic relationships with microorganisms such as fungi and bacteria (Parniske, 2008; Finkel et al., 2017). Interactions of plants with these microorganisms play an important role not only in protecting plants against various pathogens but also in defense against abiotic stresses (Lau and Lennon, 2012). Plant molecular biologists have been studying plant responses to individual environmental stresses for a long time, and attempts have been made to apply the results of these studies to agriculture. However, in the field, plants are exposed to a variety of stresses simultaneously, and the responses of plants to these stresses are often different from those predicted in the laboratory (Pandey et al., 2015). Rapid ongoing global climate change is increasing the need to study plant responses to simultaneous stresses.

The endoplasmic reticulum (ER) is one of the largest, most functionally complex, and architecturally variable organelles discovered in eukaryotic cells (Schuldiner and Schwappach, 2013). It is a highly dynamic and complex cytoplasmic membrane system composed of two structurally distinct subdomains: the nuclear envelope enclosing the nucleus and an interconnected network called the peripheral ER, comprising a series of flattened sacs and tubules (Voeltz et al., 2002; Westrate et al., 2015). The ER is a central organelle that regulates stress responses in both plant and animal cells (Ellgaard and Helenius, 2003; Schroder and Kaufman, 2005). Stresses affecting protein folding lead to ER stress, which is communicated to the nucleus *via* the unfolded protein response (UPR), a cellular homeostatic response to ER stress (Fontes et al., 1991; Ellgaard and Helenius, 2003). Although the molecular mechanism of ER stress in plants is not as well understood as in animals, the expansion and diversity of ER stress-related genes revealed by genome sequencing of various plant species suggests that plants use more ER stress responses to adapt to the environment than animals (Liu and Howell, 2010b; Howell, 2017).

**Figure 1:**
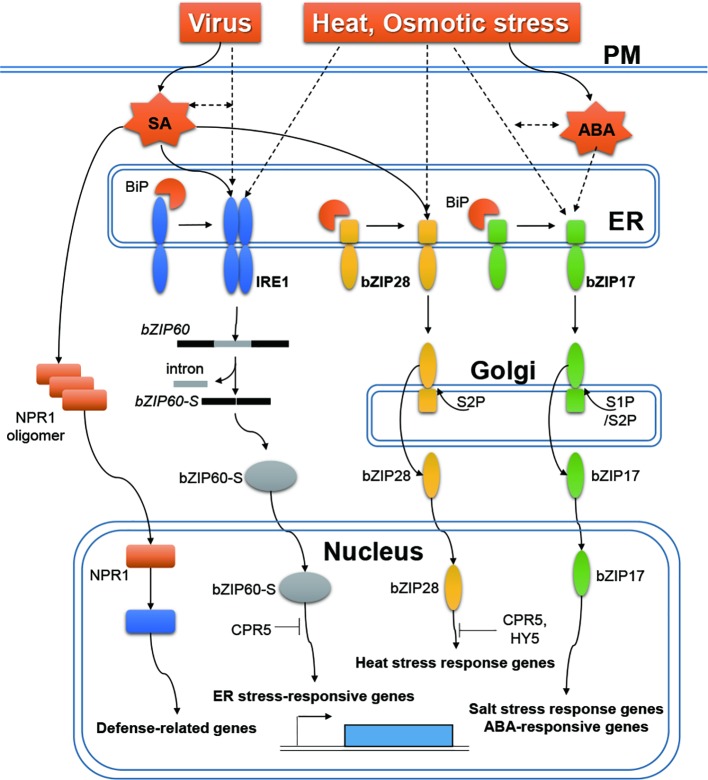
ER stress responses to RNA viruses, heat and osmotic stress, and stress-related hormones in plants. Under biotic/abiotic conditions, BiP dissociates from IRE1 to bind unfolded proteins that accumulate in the ER. Activated IRE1 splices bZIP60 mRNA (bZIP60), resulting in a frame shift that generates a bZIP60 variant (bZIP60-S) targeted to the nucleus. When BiP is sequestered away, bZIP17/28 transcription factors are released from the ER to the Golgi apparatus, where they are cleaved. The cytosol-facing regions of bZIP17/28 are transported from the Golgi apparatus to the nucleus. After translocation, bZIP transcription factors upregulate the expression of stress response genes. Expression of bZIP60 is increased by viral infection. SA induces IRE1-mediated splicing of bZIP60 mRNA and proteolytic processing of bZIP28. SA regulates the expression of stress response genes via an NPR1-depenedent pathway. ABA promotes proteolytic processing of bZIP17. Dashed arrows indicate yet-uncharacterized molecular pathways.

In plants, two main types of ER stress sensors, which regulate different UPR signaling pathways, have been identified: ER membrane-associated basic leucine zipper (bZIP) transcription factors, bZIP28 (Srivastava et al., 2014) and bZIP17 (Liu et al., 2008), and an ER resident transmembrane protein, inositol-requiring enzyme 1 (IRE1) (Koizumi et al., 2001) (Figure 1). Under unstressed normal conditions, bZIP17/28 are retained in the ER by their association with the binding protein (BiP), which is a master regulator of UPR. Under stress conditions, when unfolded proteins accumulate, BiP is sequestered away and released from bZIP17/28 (Gao et al., 2008; Srivastava et al., 2014). bZIP transcription factors are then transported from the ER to the Golgi apparatus, where they are proteolytically cleaved. The cytosol-facing regions of bZIP17/28 are then transported from the Golgi apparatus to the nucleus, to upregulate the expression of stress response genes, and to restore ER homeostasis (Liu et al., 2007a; Liu and Howell, 2010a). The IRE1 harbors both kinase and ribonuclease domains (Koizumi et al., 2001). In Arabidopsis (*Arabidopsis thaliana*), activated IRE1 splices bZIP60 mRNA, resulting in a frame shift and yielding a bZIP60 variant targeted to the nucleus (Deng et al., 2011; Hayashi et al., 2012, 2016; Liu and Howell, 2016). In the nucleus, bZIP60 plays a role in expression of ER stress response-related genes.

In this mini review, we summarize ER stress responses in plants to RNA viruses, abiotic stresses, and stress-related hormones to understand plant cell perception of simultaneous biotic and abiotic stresses and the responses to these stresses through the ER.

## Exploitation of ER Membranes by RNA Viruses

The majority of plant viruses that contains a positive-sense RNA genome and amplifies RNA in specific membrane-associated regions of host organelles are called replication complexes (RCs). Viral RCs play an important role in the enrichment of host cellular components for viral replication and prevent the activation of specific host defense mechanisms triggered by double-stranded RNA (dsRNA) intermediates of viral replication (Heinlein, 2015; Romero-Brey and Bartenschlager, 2016). Over the past decade, visualization of viral RC formation using advanced cell fluorescence imaging and microscopy techniques has enhanced our understanding of the process of virus replication in plant cells and the host factors involved (Jin et al., 2018). Although cell membrane origin and replication components and processes vary among plant RNA viruses, the RCs associated with plant RNA viruses can be divided into two types: double membrane vesicles and spherules/invaginations, as in animal viruses. This suggests that the process of viral infection is evolutionarily conserved among plant and animal viruses (Jin et al., 2018).

The ER is connected to the membrane of most other cellular organelles and the plasma membrane through membrane contact sites (MCSs) (Pérez-Sancho et al., 2016). Recent research suggests that inter-organelle communications *via* ER-driven MCSs helps communicate stress signals faster and more accurately than via vesicles or molecular diffusion (Pérez-Sancho et al., 2016). In plants, various membrane-bound organelles have been targeted as viral RC sites by one or more viral species, although the significance of interactions between these viruses and host cell organelles remains unclear (Grangeon et al., 2012). Tomato bushy stunt virus (TBSV) replicates on peroxisomes but is capable of forming viral RCs on the ER in the absence of peroxisomes, suggesting that a particular organelle is not a limiting factor for plant RNA virus infection (Jonczyk et al., 2007; Chuang et al., 2014). However, a particular type of lipid has been shown to affect viral RC formation and function. For example, the phosphatidylcholine (PC) content is high at sites of viral RCs of Brome mosaic virus (BMV), and blockade of PC synthesis inhibits BMV replication both in the native host barley and in the alternative host yeast, indicating that PC is important for BMV replication (Zhang et al., 2016). In addition, experiments using plants, yeast, and artificial phospholipid vesicles revealed that phosphatidylethanolamine is required at the site of TBSV replication (Xu and Nagy, 2015). Further research is needed to understand the effect of virus-specific lipid requirement on the host immune response, lipid metabolism in ER, and inter-organelle communication.

## ER Stress Signaling Induced by Virus Infection

Overexpression of viral proteins and changes in the lipid membrane structure during virus infection induce ER stress and UPR signaling by perturbing protein homeostasis in both plant and animal hosts (Rutkowski and Kaufman, 2004; Zhang and Wang, 2012). The importance of ER stress signaling for infection of animal viruses has been highlighted in recent years (Li et al., 2015). In particular, much work has focused on mammalian viral proteins that interact directly with components of the UPR signaling pathway to inhibit or induce this response, thereby improving viral replication or transmission in mammalian cells (Blázquez et al., 2014). More recently, increasing evidence indicates that perturbation of the lipid bilayer composition also activates UPR signaling through IRE1 and PERK independently of its effect on protein folding homeostasis in the ER lumen (Volmer et al., 2013; Halbleib et al., 2017). IRE1 and PERK mutants lacking the luminal domain, which is required to sense unfolded protein stress, are activated by deprivation of inositol in yeast and by treatment with saturated fatty acids in mammalian cells (Volmer et al., 2013; Halbleib et al., 2017). Although the molecular mechanism by which UPR sensors recognize lipid bilayer stress has not been elucidated, the hepatitis C and West Nile viruses, which belong to the family Flaviviridae, induce lipid-dependent UPR signaling through nonstructural viral proteins to facilitate viral replication and to avoid host immune surveillance (Leier et al., 2018).

The molecular components involved in ER stress and UPR signaling were identified later in plants than in animals. Consequently, associations between plant RNA virus infections and ER stress responses were mostly investigated by measuring the expression levels of ER stress-related genes such as *BiPs* and protein disulfide isomerase and calreticulin (Zhang and Wang, 2012). Overexpression of several plant viral proteins such as Turnip mosaic virus (TuMV) 6K2, Potato virus X (PVX) triple gene block protein 3 (TGBp3), Rice black-streak dwarf virus P10, and Garlic virus X p11 induces the expression of ER stress response-related genes in plants (Heinlein, 2015; Lu et al., 2016; Howell, 2017). These proteins also bind to the ER membrane and enhance expression of the *bZIP60* and *BiP* genes in tobacco (*Nicotiana benthamiana*) and Arabidopsis (Ye et al., 2011, 2012, 2013). The Arabidopsis *bZIP60* knockout mutants show a low viral titer when infected with PVX and TuMV (Zhang et al., 2015). These viruses associate with different host subcellular organelles; TuMV forms viral RCs with the peripheral membrane of peroxisomes or chloroplasts, while PVX associates with the ER. These results suggest that activation of ER stress by viral proteins through bZIP60 benefits pathogenesis of plant viruses regardless of their RC-associated subcellular organelle. In addition, eukaryotic translation elongation factor 1 alpha (eEF1A) has been known to interact with viral RNA-dependent RNA polymerase of potyviruses, tobamoviruses, and tombusviruses, which is essential for viral replication (Nishikiori et al., 2006; Yamaji et al., 2006; Thivierge et al., 2008; Li et al., 2010). A recent report demonstrated that the UPR is activated in soybean during Soybean mosaic virus (SMV) infection and that this promotes accumulation of the virus (Luan et al., 2016). The authors showed that eEF1A, which interacts with the P3 protein of SMV, plays an important role in UPR activation and viral replication, although the molecular mechanism by which eEF1A activates the UPR is unclear (Luan et al., 2016). The P3 protein is known to reside in ER as a virulence factor of SMV, but its exact biochemical function is unknown (Chowda-Reddy et al., 2011). IRE1 activates splicing of bZIP60 in plants; therefore, it would be interesting to determine whether plant viral proteins targeting ER, similar to animal viral proteins, disturb ER lipid homeostasis and utilize the ER stress response through IRE1–bZIP60 signaling to support viral RCs.

Although the mutual regulatory mechanisms of UPR and programmed cell death (PCD) are well known in animals, it is not yet clear how plant UPR is linked to pathogen-induced cell death (Shore et al., 2011). In mammalian cells, a protein kinase R (PKR)-like ER kinase (PERK) induces PCD by phosphorylating the α-subunit of eukaryotic initiation factor 2 (eIF-2α), which prevents general protein synthesis and ultimately induces apoptosis (Liu et al., 2015b). However, few studies have investigated the induction of cell death by ER stress in plants. The existence of PERK, an ER stress sensor, in plants remains controversial, and the relevance of PERK-mediated phosphorylation of eIF-2α to the general plant defense response, including that against virus infection, has not yet been clarified (Zhang and Wang, 2012). A genome-wide search for eIF-2α kinases suggests that Arabidopsis and rice (*Oryza sativa*) plants lack a mammalian PERK homolog and possess only a homolog of the yeast general control non-derepressible-2 (GCN2) (Zhang et al., 2008). Indeed, phosphorylation of eIF-2α by AtGCN2 in Arabidopsis was observed under stress conditions and unlike in animals, it was not observed during viral infection (Zhang et al., 2008). Silencing of the gene encoding p58^IPK^, a putative plant ortholog of the mammalian PKR inhibitor, causes cell death following viral infection in tobacco (Bilgin et al., 2003). In animals, p58^IPK^ acts as an inhibitor of the antiviral protein PKR, which induces apoptosis by phosphorylating eIF-2α upon recognition of viral dsRNA (Tan et al., 1998). Bilgin et al. (2003) demonstrated a direct interaction between tobacco p58^IPK^ and helicases of TMV and Tobacco etch virus and an increase in eIF-2α phosphorylation in p58^IPK^-silenced plants. These results suggest the presence of a PKR- or PERK-like kinase-mediated pathway for eIF-2α phosphorylation, although so far no PKR- or PERK-like kinases have been found in plants using sequence similarity searches with mammalian counterparts.

## ER Stress Signaling in Response to Abiotic Stress

### Heat Stress

Over the past several decades, extensive studies have elucidated several molecular mechanisms of plant responses to high temperature, mainly focusing on flower development, circadian clock modulation, and immune response (Liu et al., 2015a). Although great progress has been achieved in the identification of molecular mechanisms of plant thermotolerance, how plants sense and transduce the heat signal remains unknown. Recently, it was reported that phytochromes, which sense the ratio of red to far-red light, function as thermosensors. Indeed, phytochrome-null plants display a constitutive warm temperature response (Jung et al., 2016). In addition, elongated hypocotyl 5 (HY5), a bZIP transcription factor and a downstream component of phytochrome-mediated light signaling, negatively regulates UPR by competing with bZIP28, which upregulates the expression of stress response genes (Nawkar et al., 2017). These reports shed light on the molecular mechanism of crosstalk between UPR and thermal sensing, mediated by HY5, which positively mediates light signaling but negatively regulates UPR gene expression.

The most detrimental effect of heat shock is the accumulation of unfolded proteins in both the cytosol and ER. Therefore, unfolded protein sensors in the ER and cytosol are proposed to play an essential role in thermotolerance. One of the UPR components, bZIP28, contributes to the upregulation of heat responsive genes, leading to heat tolerance of Arabidopsis (Gao et al., 2008). Knockout mutants of bZIP28 are sensitive to high temperature, suggesting an essential role of UPR in the general heat stress response and thermotolerance (Gao et al., 2008). Chromatin immunoprecipitation coupled with high-throughput sequencing revealed 133 putative direct targets of bZIP28 in Arabidopsis seedlings subjected to heat stress (Zhang S.S. et al., 2017). Another UPR component, IRE1, is also reported to be involved in heat stress response in Arabidopsis. Heat activated IRE1 splices bZIP60 mRNA, which is required for the upregulation of BiP3 in response to ER stress (Deng et al., 2011). Furthermore, IRE1 regulates the stress transcriptome by degrading various mRNAs (Mishiba et al., 2013; Maurel et al., 2014). Therefore, phytochromes are major regulators of heat stress response and thermotolerance in plants through the adjustment of downstream molecular components and different types of ER stress sensors.

### Osmotic Stress

Osmotic stress caused by drought and high salinity has a major impact on plant growth and crop production, which highlights the importance of osmotic stress tolerance in plants. Drought and high salinity elicit many common and interactive downstream effects such as high levels of abscisic acid (ABA) (Nambara and Marion-Poll, 2005) and stress-responsive gene expression (Zhu, 2002). In Arabidopsis, the salt stress signaling pathway is reported to resemble an ER stress response (Liu et al., 2007b). Salt treatment activates the Golgi apparatus-resident site-1 protease (S1P) and cleaves ER membrane-associated transcription factor bZIP17. The released cytosol-facing region of bZIP17 is translocated to the nucleus, where it activates the expression of salt stress response genes. In this pathway, the activated bZIP17 transcription factor upregulates the expression of downstream genes, which desensitize ABA signaling (Zhou et al., 2015). Another major UPR component, BiP, plays an important role in osmotic stress tolerance in soybean (*Glycine max*) and tobacco plants *via* an unknown mechanism (Alvim et al., 2001; Valente et al., 2009). Expression profile analyses of soybean plants treated with ER stress inducers (tunicamycin/azidothymidine) or an osmotic stress inducer (polyethylene glycol) suggest a link between ER stress and osmotic stress pathway (Irsigler et al., 2007). In wheat (*Triticum aestivum*), the expression of BiP is upregulated during osmotic stress-related cell death caused by tauroursodeoxycholic acid, an apoptosis inhibitor (Zhang L. et al., 2017).

In addition to the ER-resident proteins, NAC (NAM/ATAF1/2/CUC2) domain-containing transcription factors are also receiving attention in osmotic stress response. Rice (151), soybean (152), and Arabidopsis (117) harbor a large family of NAC domain-containing proteins that are involved in multiple stress responses (Le et al., 2011; Nakashima et al., 2012; Shao et al., 2015). In soybean, GmNAC81 (also known as GmNAC6) has been identified as a component of ER stress- and osmotic stress-induced cell death response (Costa et al., 2008; Faria et al., 2011); this cell death response is synergistically activated by GmNAC30 and GmNAC81 (Mendes et al., 2013).

## ER Stress Induced by Stress-Related Hormones

### Salicylic Acid (SA)

SA is a key signaling component of both local defense response at infection sites and systemic resistance (Vernooij et al., 1994; Klessig et al., 2000). Functional crosstalk between SA and ER stress was first observed in Arabidopsis (Wang et al., 2005). Treatment of Arabidopsis with SA alters the level of many ER proteins required for protein folding and secretion, including BiP2 (Wang et al., 2005) and BiP3 (Pajerowska-Mukhtar et al., 2012). SA-induced expression of some of the ER stress-related genes is regulated by the heat shock factor-like transcription factor, *TL1*-binding transcription factor 1 (TBF1), which is genetically dependent on the non-expressor of pathogenesis-related genes 1 (NPR1), a master regulator of SA signaling (Wang et al., 2005; Pajerowska-Mukhtar et al., 2012). Because TBF1 regulates only selected ER genes, it was initially suggested that plants have evolved a specific mechanism for regulating SA-induced expression of ER stress-related genes (Pajerowska-Mukhtar et al., 2012). It was later shown that SA induces these genes *via* two main UPR signaling pathways: proteolytic processing of bZIP28 and IRE1-mediated splicing of bZIP60 mRNA (Nagashima et al., 2014). However, because it is unlikely that SA directly inhibits protein folding in the ER, the mechanism of activation of the UPR pathway by SA remains unclear. In Arabidopsis, constitutive expresser of pathogenesis-related genes 5 (CPR5), a plant-specific growth and stress regulator, acts as a negative modulator of SA in the early signal transduction steps, downstream of pathogen recognition and upstream of SA (Bowling et al., 1997). Recently, CPR5 was reported to act as a negative regulator of bZIP28 and bZIP60 through protein–protein interactions (Meng et al., 2017). Consequently, CPR5 suppresses the function of ER stress-induced bZIP28 and IRE1-bZIP60 pathways in the homeostatic control of SA-mediated plant growth (Meng et al., 2017).

### ABA

ABA regulates many aspects of plant growth and development and plays a central role in the response to heat and osmotic stress (Shinozaki and Yamaguchi-Shinozaki, 2000; Raghavendra et al., 2010; Hauser et al., 2011; Huang et al., 2016). For example, in Arabidopsis, high temperature treatment upregulates ABA biosynthesis-related genes and downregulates ABA degradation-related genes (Toh et al., 2008). Similarly, in rice, heat treatment increases the level of ABA (Wu et al., 2016). Despite the increasing number of reports on ABA and heat stress, only a few studies have been conducted to investigate the effect of ABA on ER stress. Arabidopsis plants overexpressing the maize ortholog of Arabidopsis bZIP17 (ZmbZIP17) upregulates the expression of ER stress response genes. ER stress inducers such as dithiothreitol and tunicamycin induce ZmbZIP17 and its translocation to the nucleus (Yang et al., 2013). In addition, ZmbZIP17 interacts with ABA-responsive cis-elements in the promoter of ABA-responsive genes in yeast. Considering these reports, it is very likely that bZIP17 is involved in ABA-mediated ER stress response.

The mechanism of activation of bZIP17, which triggers ABA signaling in response to ER stress, has been elucidated in a study on seed germination in Arabidopsis; the authors showed that the Golgi apparatus-resident site-2 protease (S2P) cleaves and activates bZIP17, thus regulating downstream target genes that encode negative regulators of ABA signaling (Zhou et al., 2015). The level of ABA is also elevated by salt stress, which induces a signaling cascade involving the processing of bZIP17 by S1P, translocation of bZIP17 to the nucleus, and upregulation of salt stress genes (Nambara and Marion-Poll, 2005; Liu et al., 2007b). However, whether bZIP17-mediated ABA response is directly linked to the ER stress response remains unclear. It is possible that salt or ABA treatments promote protein misfolding, and S1P/S2P-mediated bZIP17 processing plays an essential role in desensitizing the plant to ER stress.

## Conclusion and Perspectives

Changes in the agricultural environment, such as changes in temperature and water availability caused by climate change, can cause enormous reductions in crop yields *via* increased biological stress (Pandey et al., 2017). Global warming, for example, increases temperature stress in plants while at the same time increasing insect populations, which results in the spread of insect-borne viruses and their expansion to new host areas (Bebber, 2015). To help crops withstand newly emerging stresses, it is important to understand the mechanisms that plants have evolved to counteract various stress factors. Here, we have summarized recent advances in our understanding of ER responses to RNA viruses, abiotic stresses, and hormone responses (Figure 1). Of the three known ER stress sensors, IRE1 and bZIP28 are both involved in ER stress responses to viral infection and abiotic stress, whereas bZIP17 appears to be only an abiotic stress-specific sensor. Prasch and Sonnewald (2013) have shown that Arabidopsis plants exposed simultaneously to multiple stresses, such as heat, drought, and TuMV infection, have different responses to those of plants exposed to only one stress. For instance, defense-related genes induced by viral infection were not observed in Arabidopsis plants exposed to three stresses, but the ER stress response, which was not observed during TuMV infection, was induced (Prasch and Sonnewald, 2013). These results suggest that the mechanisms by which plants adapt to both external and internal stresses must be elucidated to understand the molecular adaptation of plants to multiple external stresses.

## Author Contributions

JMP designed the outline of the article and C-JP and JMP wrote the article.

### Conflict of Interest Statement

The authors declare that the research was conducted in the absence of any commercial or financial relationships that could be construed as a potential conflict of interest.
